# Lipid deposition pattern and adaptive strategy in response to dietary fat in Chinese perch (*Siniperca chuatsi*)

**DOI:** 10.1186/s12986-018-0315-6

**Published:** 2018-11-01

**Authors:** Jie Wang, Xu-Fang Liang, Shan He, Jiao Li, Kang Huang, Yan-Peng Zhang, Dong Huang

**Affiliations:** 1 College of Fisheries, Chinese Perch Research Center, Huazhong Agricultural University, No.1, Shizishan Street, Hongshan District, Wuhan, 430070 Hubei Province China; 2 Freshwater Aquaculture Collaborative Innovation Center of Hubei Province, Key Lab of Freshwater Animal Breeding, Ministry of Agriculture, Wuhan, 430070 China

**Keywords:** Chinese perch, Dietary fat, Lipid homeostasis, Lipogenesis, FAs β-oxidation, Gluconeogenesis, Hepatic steatosis

## Abstract

**Background:**

Previous studies in teleost have demonstrated the adaptive strategy to maintain hepatic lipid homeostasis within certain limit. The excess of fat-intake could induce abnormal lipid deposition in liver but not adipose tissue. However, the molecular mechanism between the impaired lipid homeostasis and the aggravated lipid deposition in liver has not been elucidated well in fish.

**Methods:**

Four isonitrogenous diets with different fat levels (2, 7, 12 and 17%) were formulated, named L2, L7, L12 and L17 respectively, and fed Chinese perch (44.50 ± 0.25 g) to apparent satiation for five weeks. Growth index, triglyceride concentrations and expression of genes involved in lipid metabolism were measured.

**Results:**

The maximal growth performance and food intake were observed in L12 group. The lipid content in liver and serum were comparable in L2, L7 and L12 groups, while they were increased significantly in L17 group. Histology analysis also demonstrated that mass lipid droplets emerged in hepatocyte and then induced hepatic steatosis in L17 group. Compared to L2 group, the lipolytic genes related to fatty acids (FAs) transport (*lpl* & *hl*) and FAs β-oxidation (*cpt1* & *cs*) were increased in L7 and L12 group. Relative mRNA levels of the gluconeogenesis (*pc*, *pepck* & *g6pase*) were also increased, in contrast, the lipogenic genes (*srebp1*, *accα* & *fas*) were decreased. Compared to L12 group, L17 group had higher mRNA levels of the FAs transport and the lipogenesis. But the lipolytic genes related to FAs β-oxidation were steady and the mRNA levels of gluconeogenesis were down-regulated instead.

**Conclusions:**

Within certain limit, the increase of dietary fat in L7 and L12 group was propitious to reduce the consumption of protein and improve growth performance in Chinese perch. It was due to the homeostasis of hepatic triglyceride (TG) pool and serum glucose through promoting the FAs β-oxidation and gluconeogenesis respectively. Both the increase of lipogenesis and the absence of FAs β-oxidation in L17 group could trigger the esterification of FAs, indeed, the inhibition of gluconeogenesis could also aggravate triglyceride accumulation in liver and induce hepatic steatosis.

## Background

Dietary fat could provide high energy and essential fatty acid (EFA) to satisfy the rapid growth rate and the requirement of physiological lipid in most cultured fishes [1–5]. Within certain limits, the increase of dietary fat level could improve the utilization of feed [6] and protect somehow against the metabolism of protein for energy [7, 8], especially in carnivorous fish species. However, high fat diets led to the increased fat deposition in fish body, induced metabolic impairments including fatty liver syndrome [9], abnormal oxidative status [10], and altered nutritional value, organoleptic and physical properties [6]. Many research findings revealed that the variation of dietary fat level must be carefully evaluated as it may affect lipid metabolic strategies and lipid deposit pattern [2, 11–17].

Although different fish species have different tolerance on exogenous fat-intake, natural selection endows fish with the abilities to store lipid in different organs when dietary fat is abundant, on the other hand, it accelerates lipid mobilization for providing energy. Generally, with the increasing dietary fat-intake, the lipid content of fish body was also increased progressively [6, 16, 18–21]. Interestingly, the priority site of fat deposition was species-specific in fish. Several fish species stored lipid (mainly TG) in mesentery and viscera preferentially, like grass carp (*Ctenopharyngodon idella*) [10, 11, 14, 17] and Nile tilapia (*Oreochromis niloticus*) [12], while Atlantic salmon (*Salmo salar*) would prefer storing lipid in muscle but not liver [13]. The selective strategy could contribute to reduce the potential risk of fat deposits in liver. Besides, the homeostasis of hepatic lipid could depend on the hepatic mitochondrial and peroxisomal oxidation capacities. Because the activation of hepatic mitochondrial oxidation could accelerate the degradation of free fatty acids (FFAs) via carnitine palmitoyltransferase I (CPT1) and then release adenosine triphosphate (ATP) for providing energy through the tricarboxylic acid (TCA) cycle [22].

It was noticed that a sustained high-fat intake could easily impair lipid homeostasis and consistently induce the mass accumulation of TG in abnormal sites [23]. In mammal models, animals fed with high-fat diets would usually store lipid not only in white adipose tissue (WAT), a specific lipid deposit organ mainly composed of adipocytes, but also in other abnormal sites, such as liver, skeletal muscle, and even pancreatic β-cells, kidney [24]. While in some teleost’s models, such as white seabass (*Atractoscion nobilis*) [15], Japanese seabass (*Lateolabrax japonicus*) [25], haddock (*Melanogrammus aeglefinus* L) [26], and turbot (*Psetta maxima*) [19], the abnormal site of TG deposition was liver primarily in response to high-fat intake. Lipid deposition in abnormal sites could be emerged in three pathways: increased uptake of FAs, increased synthesis within the tissue involved and/or reduced FAs oxidation/disposal [27]. When it comes to excess fat-intake in organism, liver plays a critical role in lipid transport, lipid catabolism (mainly lipolysis and FAs b-oxidation), lipogenesis and even lipid deposition [28, 29]. Indeed, mass circulating FFAs in the blood could be absorbed into liver for esterification via lipogenesis and then repackaged with TG form, finally, delivered into extrahepatic tissue via TG-rich lipoprotein (mainly very low-density lipoprotein, VLDL). In briefly, the ectopic accumulation of TG in liver was due to an imbalance between lipid availability from circulating lipid uptake or lipogenesis and lipid disposal via FAs β-oxidation or TG-rich lipoprotein secretion [30].

Chinese perch (*Siniperca chuatsi*), as a typical carnivorous freshwater fish, is one of the economical cultivated fish species in China as well in some other Asian countries [31]. Chinese perch was treated as a suitable fish model for the fundament study of nutrition and metabolism because of its faster growth rate and higher tolerance to severe survival conditions [32]. However, Chinese perch owns a peculiar feeding habit, only after acclimation with a standard training protocol, could they accept artificial diets smoothly [33]. Therefore, compared with the other cultivated freshwater fishes, Chinese perch need extra protein to satisfy its better growth performance, and the requirement of dietary protein was up to 47% [33]. So how to reduce the investment of protein by using lipid is a mentionable issue in cultivation of Chinese perch. Excess dietary fat, on the other hand, would also cause fatty degeneration, hepatic inflammation and fatty liver syndrome, and then caused an adverse effect on its health and carcass quality. Thereby, clarifying the inner mechanisms of lipid metabolism and deposition is an urgent issue based on the requirement of healthy aquaculture production and better nutritionists of Chinese perch. The hypothesis of this study was that Chinese perch could be possessed with the adaptive ability to maintain hepatic lipid homeostasis though selecting the priority site of TG deposit and accelerating lipid catabolism in response to dietary fat level with certain limits. But when it comes to high-fat diets, mass newly-synthesized TG could accumulate in liver primarily based on lipid metabolic disorders via the absence of lipid mobilization. To that end, the present study was conducted the effects and mechanism of dietary fat levels influencing growth performance and lipid metabolism.

## Methods

### Ethical approval

All experimental procedures followed the guidance for animal protocol and were approved by Huazhong Agricultural University (Wuhan, China).

### Animals and feeding

Chinese perch were obtained from Wuhu agricultural development company and cultured in the fish house of Huazhong Agricultural University (Wuhan, China). Prior to experiment, all the fish would accept the artificial diets after acclimation [34]. Four diets were formulated with different gradient lipid (2, 7, 12 and 17%, respectively) and coded as L2, L7, L12, and L17 group (Table 1). All the dietary ingredients were purchased from Gaolong Feed Technology Co., Ltd. (Wuhan, China). Then the total 144 fishes were selected and arranged randomly into 12 tanks (350 L) with a constant flow of filtered water. The stocking density was 12 fishes (44.50 ± 0.25 g fish^− 1^) per tank and each diet was arranged to triplicate tanks. During the period of culture, all the fish were fed twice a day at 8.30 am and 17.30 pm to apparent satiation. The water temperature was maintained at 24 ± 2 °C, and the water was changed twice a week by using circulating water system.

**Table 1:** Compositions of diets added with different levels of lipid

Experimental diets	L2	L7	L12	L17
Ingredients (g kg^− 1^)				
Fish meal	745	695	645	590
Casein^a^	11	48	85	126
Fish oil^b^	0	25	50	75
Soybean oil^c^	0	25	50	75
Corn starch^d^	60	60	60	60
Cellulose	134	97	60	24
Mineral mix^e^	20	20	20	20
Vitamin mix^f^	20	20	20	20
Carboxymethylcellulose sodium	10	10	10	10
Total	1000	1000	1000	1000
Proximate composition				
Dry matter (DM) (%)	81.38	86.75	89.12	93.52
Crude protein (% DM)	47.58	47.52	47.55	47.51
Crude lipid (% DM)	2.32	7.72	12.44	17.15
Carbohydrate (% DM)	13.39	13.36	13.40	13.42
Ash (% DM)	17.48	18.13	15.77	15.44
Gross energy (kJ g^−1^)	1.23	1.41	1.59	1.77

^a^Crude protein and crude lipid content of casein was 84.4 and 0.6%, respectively

^b^Fatty acids composition of fish oil (%): 14:0, 10.01; 16:0, 15.61, 16:1, 10.25; 18:0, 1.63; 18:1, 11.88; 18:2, 4.29; 18:3, 6.20; 20:0, 0.07; 20:4, 3.80; 20:5 (EPA), 19.10; 22:6 (DHA), 7.69; SFA, 27.32; UFA, 63.21; PUFA, 41.08; MUFA, 22.13

^c^Fatty acids composition of soybean oil (%): 10:0, 0.01; 14:0, 0.03; 16:0, 12.04; 16:1, 0.07; 18:0, 1.81; 18:1-9c, 28.77; 18:1-9 t, 0.01; 18:2-9c12c, 55.57; 18:2-9t12t, 0.02; 20:0, 0.39; 20:1, 0.20; 20:2, 0.01; 22:0, 0.11; 23:0, 0.02; 24:0, 0.01; 24:1, 0.04; SFA, 14.69; UFA, 85.29; MUFA, 29.12; PUFA, 56.16

^d^Crude protein and crude lipid content of corn starch was 0.3 and 0.2%, respectively

^e^Mineral premix (per kg of diet): MnSO4, 10 mg; MgSO4, 10 mg; KCl, 95 mg; NaCl, 165 mg; ZnSO4, 20 mg; KI, 1 mg; CuSO4,12.5 mg; FeSO4, 105 mg; Na2SeO3, 0.1 mg; Co, 1.5 mg

^f^Vitamin premix (per kg of diet): vitamin A, 2000 IU; vitamin B1 (thiamin), 5 mg; vitamin B2 (riboflavin), 5 mg; vitamin B6,5 mg; vitamin B12, 0.025 mg; vitamin D3, 1200 IU; vitamin E 21 mg; vitamin K3 2.5 mg; folic acid, 1.3 mg; biotin, 0.05 mg; pantothenic acid calcium, 20 mg; inositol, 60 mg; ascorbic acid (35%), 110 mg; niacinamide, 25 mg

### Samples collection and chemical analyses

After 5 weeks feeding trial, all the fishes were starved for 24 h and euthanized with MS-222 (Argent Chemical Laboratories, Redmond, WA, USA), and then weighted and counted. For each treatment, six fishes were randomly captured and stored in a freezer at − 20 °C until used for the whole-body chemical analysis. Blood was taken from the caudal vein of six fishes in each group and stored at 4 °C overnight, then centrifuged (2500 g, 20 min) for serum samples. Serum samples were frozen at − 80 °C until analysis. Tissue samples for lipid contents detection, like liver, mesentery and visceral adipose, were dissected from six fishes in each treatment and then stored at − 20 °C. Hepatic somatic index (HSI), mesentery fat index (MFI) and visceral somatic index (VSI) were calculated immediately after anatomy [HSI = the weight of liver / body weight (%), VSI = the weight of visceral adipose tissue / body weight (%) and MFI = the weight of mesenteric fat / body weight (%)]. Another six fishes per treatment were randomly chosen for molecular experiments, and liver tissue (0.5 g) for genes expression assay were fast frozen in liquid nitrogen and then stored at − 80 °C for RNA isolation and subsequent analysis.

The chemical analyses including dietary and whole-body composition analyses were determined by standard methods [35]. The moisture were analyzed by drying at 105 °C for 6 h. The determination of crude protein (N × 6.25) was conducted by using the Kjeltec system after acid digestion (K8400 Kjeltec Analyzer, Fossana Lyticab, Sweden). The crude lipid was measured by using the ether-extraction with Soxtec System HT (SE-A6, Alvah, China). Ash was determined by combustion with muffle furnace (SX2–4-10, Zhengda Electric Technology Co., Ltd., China) at 550 °C for 12 h.

The biochemical analyses were focused on the detection of serum indices including GLU (glucose), TC (total cholesterol), TG (total triglyceride), HDL (high-density lipoprotein), LDL (low-density lipoprotein) and AST (aspartate aminotransferase). All of these indices were determined with an automatic biochemical analyzer [Abbott Aeroset Analyzer (Abbott Laboratories, USA)] in Zhongnan Hospital (Wuhan, China).

### Histology analysis and reagents

Liver tissues from three fishes from each group were collected and immediately fixed by using 4% neutral buffered formaldehyde for 4 days. After dehydrated and imbedded into paraffin, a tissue section was cut into 5 μm for hematoxylin and eosin (H&E) staining (Catalog no. G1005–100; Servicebio Biotech Technology Co., Ltd., Wuhan, China). For the frozen section and Oil-red O staining, liver samples were immediately frozen with liquid nitrogen and stored at − 80 °C. Serial frozen sections were cut into 8 μm for Oil-red O staining. The dried slides are washed by dipping them one or two times in 70% alcohol, and they are then placed on an absorbent surface and covered with the Oil Red O solution (Catalog no. G1016; Servicebio Biotech Technology Co., Ltd., Wuhan, China) for 5 min. The liver sections were viewed at 40× magnification. Three slides from each group were included in quantification and 5 fields were randomly selected on each slide. For each field, the number of cell nuclei in the H&E observation and the relative area of lipid droplet in Oil Red O observation were quantified by Image J software (National Institutes of Health) following previous reports [36, 37]. The TG contents in liver, visceral adipose tissue and muscle were performed by the manufacturer of Triglycerides Assay Kit (Catalog no. F001; Jiancheng Bioengineering Institute, Nanjing, China).

### RNA isolation and reverse transcription

Prior to RNA isolation, liver tissues were taken out from the - 80 °C fridge and unfrozen on the ice. Trizol reagent (Code no. 9108; TaKaRa, Japan) was use as a lysis buffer for liver tissues, added 1 mL with 0.1 g liver sample. Followed by the manufacture’s instruction, RNA is purified by phenol/chloroform extraction of the lysate supernatant followed by ethanol precipitation. The extracted RNA was dissolved in 50–100 μL RNase-free water (Code no. 9750; TaKaRa, Japan). The RNA integrality was examined with agarose gel electrophoresis. The concentration of RNA samples was quantified with a BioTek Synergy™ 2 Multi-detection Microplate Reader (BioTek Instruments, USA). Then 1 μg of total RNA was used for reverse transcription with HiScript® II Reverse Transcriptase (Code no. R201–01/02; Vazyme, China) in a 20 μL reaction volume. The synthesized cDNA was stored at − 20 °C until further use.

### Real-time qPCR analysis

The quantification of genes was performed with RT-PCR analysis. All the gene-specific primers are presented in Table 2, and the sequences were obtained from our previous transcriptome sequencing of Chinese perch [38]. Followed by designing primers on Primer 5.0 software, all the primers lists were sent to Sangon Biotech Company (Shanghai, China) for synthesizing. The specificity of the primers was determined through sequencing, and the melting curve of PCR products. Plasmid containing target fragments were dilated 10-fold and qPCR was conducted using different dilutions as templates to construct standard curves for genes. The amplification efficiencies were analyzed according to the slope of the standard curve in a given run. Moreover, several housekeeping genes containing *β-actin*, *rpl13a*, *b2m*, *ywha2*, *hmbs* and *sdha* were chosen based on the published article [39]. After comprehensive comparison of the candidate housekeeping genes, the *rpl13a* gene expression was more stable to apply as an internal reference. All amplifications for each sample were determined by MyiQ™ 2 Two-Color Real-time PCR Detection System (BIO-RAD, USA). The reaction volume was containing 10 μL 1 × AceQ® qPCR SYBR® Green Master Mix (Code no. Q121–02/03; Vazyme, China), 0.4 μL of each primer and 1 μL cDNA. And the PCR cycling parameters were 95 °C for 5 min followed by 40 cycles at 95 °C for 10 s, annealing temperature for 30 s and a melt curve step from 65 °C, gradually increasing from 0.5 °C/s to 95 °C, with acquisition data every 6 s. The technical error was excluded by performing, in triplicate of each sample. Gene expression levels were quantified relative to the expression of *rpl13a* using the optimized comparative Ct (2^-ΔΔCt^) value method [40]. All data are presented with mean ± S. E.M. (*n* = 6).

**Table 2:** Primer sequences for the quantitative real-time qPCR

Gene name	Sequence 5′-3′	Tm (°C)	Product size (bp)	E-values (%)
*rpl13a*	CACCCTATGACAAGAGGAAGC	59	100	102.9
	TGTGCCAGACGCCCAAG			
*pepck*	CTGAGTTTGTGAAGAGAGCGG	57	170	100.3
	GTCCTTTGGGTCTGTGCGT			
*srebp1*	CTCCCTCCTTTCTGTCGGCTC	58	111	103.2
	TCATTTGCTGGCAGTCGTGG			
*fas*	ATGGAAATCACCCCTGTAATCTT	57	203	101.9
	CTTATCTGACTACGGAATGAATCG			
*accα*	TATGCCCACTTACCCAAATGC	58	129	102
	TGCCACCATACCAATCTCGTT			
*cpt1*	ATGGTGTATTGGCTGGAGTCT	57.5	139	102.8
	CTGTGTGGTAGGTTTTCCTTGAT			
*cs*	GAATGCCACCTACTTCCTTGT	57	166	98
	CCCCTCATACCTCCATAAACC			
*pc*	GTCCCGTTCCAGATGC	54	257	101
	CTGCCAGTTTCAGATAGTAGTCC			
*g6pase*	TGTGGATGGCTTTTTGGGT	58	342	101.5
	CAGAGTGAGTGGGCATTTTGAT			
*apoe*	TGAGCGACATTTCCACCATA	57	267	95.8
	CACCAACCAACTACAACCCAT			
*lpl*	TTACCCCAATGGAGGCACTT	58	277	98.8
	CGGACCTTGTTGATGTTGTAG			
*hl*	CAACCCTGAAGACAAATCTAATA	57.5	180	96.3
	CAATCAAATGAGCCTTTCTAACT			

### Statistical analysis

Statistical analyses were performed with SPSS 19.0 software. The normality and homogeneity of variances for all data were respectively assessed by Shapiro-Wilk’s test and Levene’s test. The means differences were tested by Duncan’s multiple range tests with one-way analysis of variance (ANOVA), statistical significance was considered to be at the 5% level.

## Results

### Growth performance and feed utilization

After 5 weeks feeding trial, the feed utilization and growth performance were presented in Table 3. From L2 to L12 group, the food intake (FI) showed no significant difference, but the final weight (FW), weight gain (WG), specific growth ratio (SGR) and protein retention (PR) were progressively elevated. All of the above indices were decreased markedly in L17 group compared to L12 group.

**Table 3:** Growth performance and feed utilization of Chinese perch

Item	L2	L7	L12	L17
IW	44.52 ± 0.04	44.31 ± 0.06	44.59 ± 0.12	44.73 ± 0.05
FW	56.07 ± 0.51^a^	61.86 ± 0.21^c^	65.03 ± 0.49^d^	60.00 ± 0.39^b^
WG (%)	25.93 ± 1.03^a^	39.58 ± 0.66^c^	45.84 ± 1.45^d^	34.15 ± 0.66^b^
SGR (%)	0.66 ± 0.02^a^	0.95 ± 0.01^c^	1.07 ± 0.03^d^	0.84 ± 0.01^b^
FI (g fish^−1^)	533.55 ± 32.45^bc^	563.46 ± 20.32^c^	585.94 ± 24.16^c^	468.22 ± 17.80^ab^
PR (%)	23.50 ± 0.41^a^	36.15 ± 0.64^c^	42.22 ± 0.26^d^	26.49 ± 0.53^b^

Values are means ± SEM of four replicates, and values within the same row with totally different letters in superscript are significantly different (*P* < 0.05). IW (g), initial weight; FW (g), final weight; Weight gain (WG, %) = 100 × (final weight – initial weight) / initial weight; Specific growth ratio (SGR, %) = 100 × (ln FW – ln IW) / time (days); FI (g fish^− 1^), food intake; PR (%) = (fish protein gain) × 100 / (protein intake)

### Effects of dietary fat levels on lipid depositional sites

**Fig. 1:**
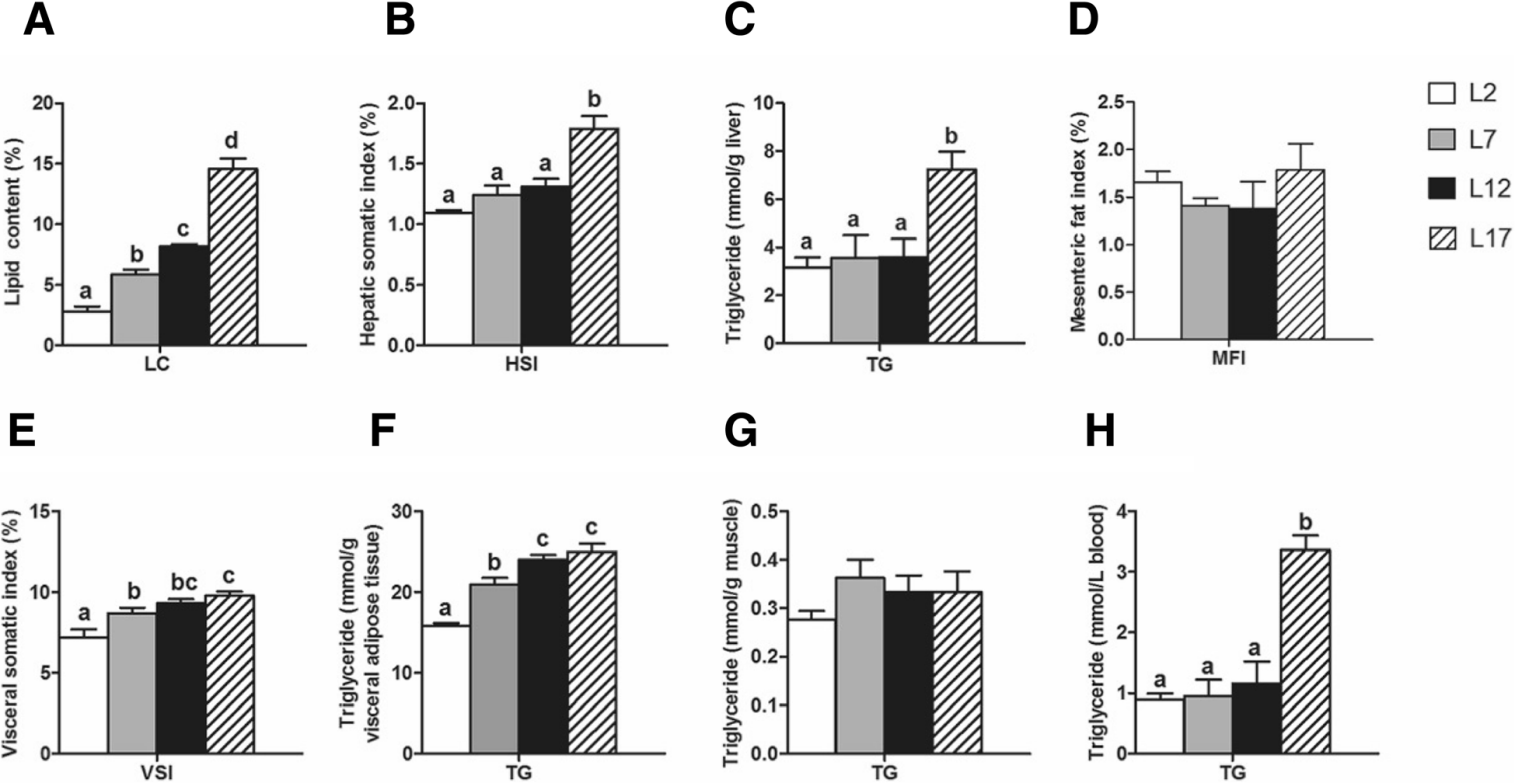
Lipid distribution of different tissues in Chinese perch. **a** The crude lipid content of whole fish body. **b** Hepatic somatic index. **c** TG content in liver. **d** Mesenteric fat index. **e** Visceral somatic index. **f** TG content in visceral adipose tissue. **g** TG content in muscle. **h** TG content in blood. For A-H, Values are means ± SEM (*n* = 6). Values with totally different letters on columns statistically differ at *P* < 0.05 and values without letters on columns mean no difference (one factor ANOVA, Duncan’s post hoc test)

The lipid content (LC) of fish body was progressively elevated with the increased dietary fat from 2 to 17% (Fig. 1a). The MFI and the TG content in muscle showed no difference among the four groups (Fig. 1d and g). Compared to L2 group, the VSI and the TG content in visceral adipose tissue were increased markedly in both L7 and L12 group (Fig. 1e, f). The HSI and the TG content in liver and serum showed no difference among L2, L7 and L12 group (Fig. 1b, c and h). Compared to L12 group, the HSI and the TG content in liver and serum were raised significantly in L17 group, but the VSI and the TG content in visceral adipose tissue were stable.

### Effects of dietary fat levels on serum indices

Serum TC, HDL, LDL and GLU concentrations were shown in Table 4. Serum glucose concentration was increased progressively from L2 to L12 group, but decreased sharply in L17 group. The serum TC, LDL and HDL were increased markedly in L17 group compared to other groups.

**Table 4:** Serum lipid fractions of Chinese perch

Item	L2	L7	L12	L17
GLU	1.99 ± 0.18^a^	3.42 ± 0.49^b^	4.68 ± 0.60^c^	2.16 ± 0.11^a^
TC	5.12 ± 0.39^b^	5.24 ± 0.63^b^	4.59 ± 0.40^b^	7.75 ± 0.35^a^
HDL	0.80 ± 0.04^ab^	0.88 ± 0.05^ab^	0.96 ± 0.09^b^	1.21 + 0.05^c^
LDL	0.71 ± 0.09^b^	0.83 ± 0.10^b^	0.69 ± 0.16^b^	1.26 ± 0.07^a^

Values are mean ± S.E.M of six replicates and values within the same row with different letters are significantly different (*P* < 0.05). GLU, glucose; TC, total cholesterol; TG, total triglyceride; HDL, high-density lipoprotein; LDL, low-density lipoprotein

### Effects of dietary fat levels on hepatic tissue section

**Fig. 2:**
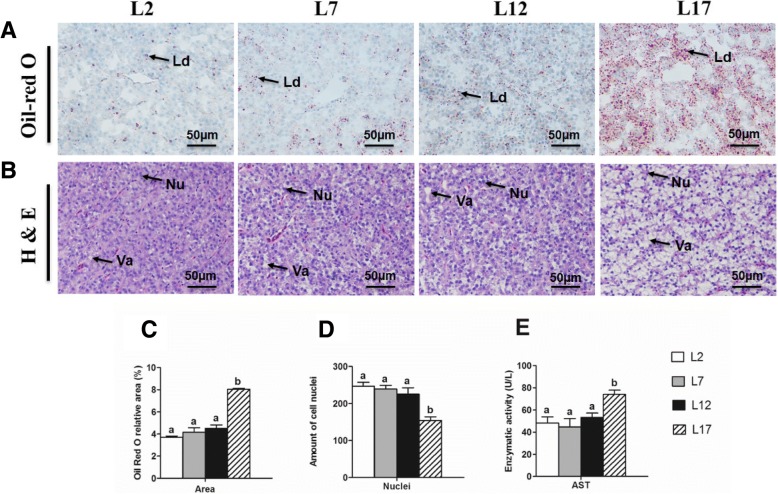
Histology analyses of liver section and determination of hepatocyte inflammation. **a** Hepatic tissue section (40× magnification) of Oil Red O staining of Chinese perch fed with 2, 7, 12 and 17% dietary lipid levels. **b** Hepatic tissue section (40× magnification) of hematoxylin and eosin staining of Chinese perch fed with 2, 7, 12 and 17% dietary lipid levels. **c** Oil Red O relative area. **d** Measurement of hepatic cell nuclei. **e** Blood aspartate aminotransferase. Lipid droplets appear red after staining Oil Red O, and the depth of color of the red stain and the amount of the lipid droplet were positively correlated with lipid content. The nuclei of hepatocyte appear blue and the vacuole present to be hyaline after staining hematoxylin and eosin, and the numbers of nuclei were negatively correlated with hepatic steatosis. Ld, lipid droplet; Nu, nuclei; Va, vacuole. For C and D, Values are means ± SEM (*n* = 3); for E, Values are means ± SEM (n = 6). Values with totally different letters on columns statistically differ at *P* < 0.05 (one factor ANOVA, Duncan’s post hoc test)

Excessive fat intake was usually correlated with the ectopic TG deposition in liver. In order to investigate whether high-fat diets could induce hepatic steatosis in Chinese perch, we performed Oil-red O staining and H&E staining on liver sections (Fig. 2). The Oil-red O staining confirmed that the number of red dots (lipid droplets) has exhibited no obvious difference among L2, L7 and L12 group, but it increased sharply in L17 group (Fig. 2a). Indeed, the H&E staining showed that liver cells appeared small vacuoles progressively from L2 group to L12 group, but in L17 group, the vacuole was enlarged sharply and then squeezed the cell nuclei to the edge (Fig. 2b). These results were further confirmed by the quantified area for lipid droplets in the Oil-red O staining and by the number of cell nuclei in the H&E staining. The relative area of lipid droplet in L17 group was significantly larger than other groups, on the other hand, the number of cell nuclei was descended only in L17 group (Fig. 2c, d). That means liver cells had suffered from pathological reaction which was induced by TG infiltration. In addition, the AST enzymatic activity in blood, a maker of hepatic injury, was significantly elevated in L17 group compared with other groups (Fig. 2e).

### Genes expression of glucose and lipid metabolism in liver

**Fig. 3:**
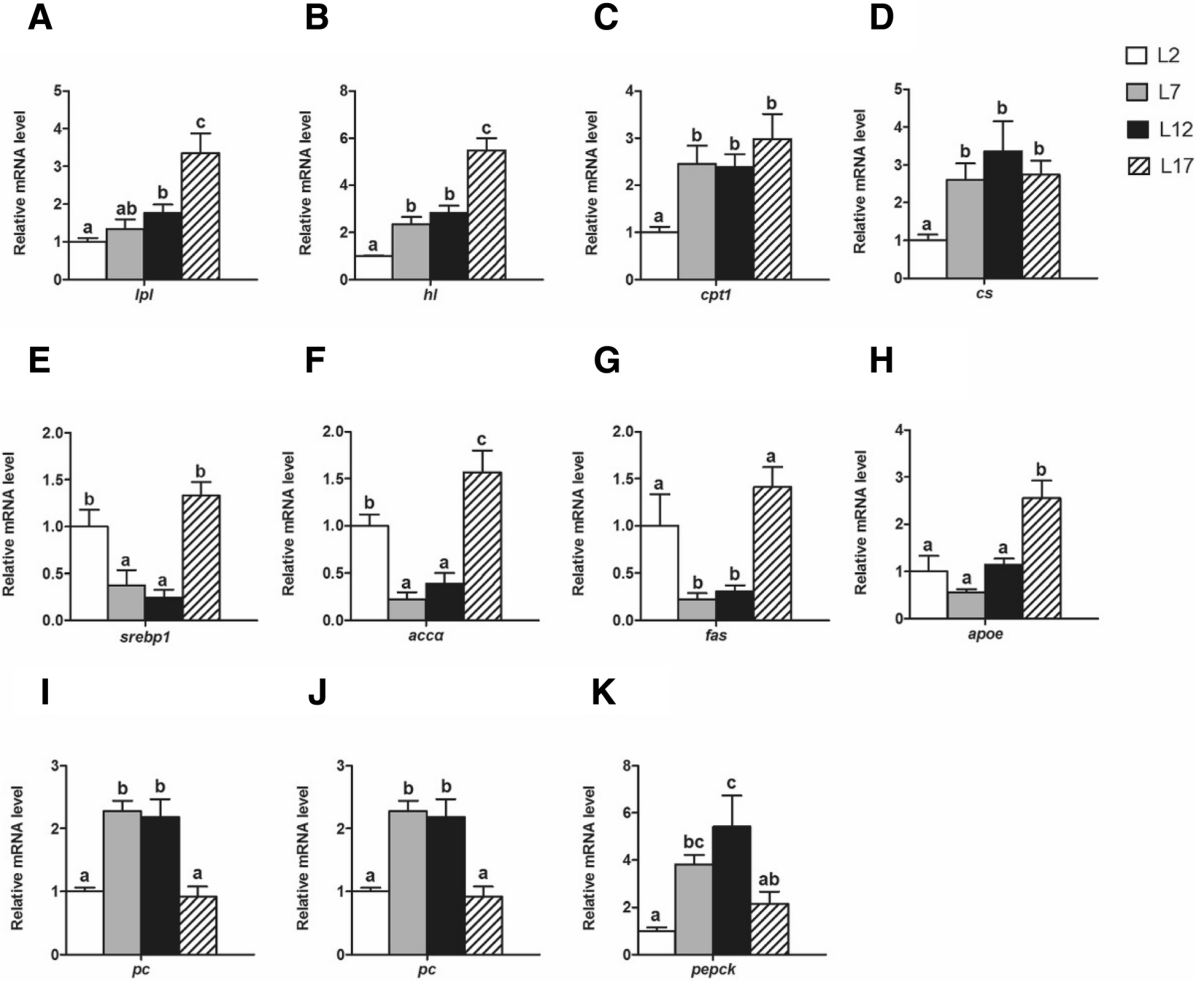
The mRNA expression of the genes in hepatic triglyceride metabolism of Chinese perch. **a-b** The relative mRNA levels of *hl* and *lpl* were related to the triglyceride hydrolysis and hepatic FAs transport. **c** The relative mRNA abundance of *cpt1* was related to FAs β-oxidation. **d** The relative mRNA level of *cs* was related to the conversion from acetyl-CoA and oxaloacetate into citrate. **e** The relative mRNA abundance of *srebp1* was related to lipogenesis in liver. **f-g** The relative mRNA levels of *accα* and *fas* were related to FAs biosynthesis. **h** The relative mRNA abundance of *apoe* was related to triglyceride transport. **i-k** The relative mRNA levels of *pc*, *pepck* and *g6pase* were related to gluconeogenesis de novo. For A-K, Values are means ± SEM (n = 6). Values with totally different letters on columns statistically differ at *P* < 0.05 (one factor ANOVA, Duncan’s post hoc test)

Some relative genes expression, which were involved in the lipid and glucose metabolism in liver of Chinese perch, were analyzed by real-time qPCR (Fig. 3). The relative mRNA levels of lipoprotein lipase (*lpl*) and hepatic lipase (*hl*), the key regulator of TG hydrolysis and FAs transport in liver, were increased progressively from L2 to L17 group (Fig. 3a, b). The relative mRNA levels of carnitine palmitoyltransferase I (*cpt1*) and citrate synthase (*cs*), the two major rate-limiting enzyme participated in FAs β-oxidation and TCA circle respectively, were elevated significantly in L7 and L12 group, but it showed no difference between L12 and L17 group (Fig. 3c, d). The expression of lipogenic genes, including in sterol regulatory element binding protein 1 (*srebp1*), acetyl-CoA carboxylase alpha (*accα*) and fatty acid synthase (*fas*), were sharply down-regulated both in L7 and L12 group, and then up-regulated in L17 group (Fig. 3e, f and g). The overexpression of Apo-lipoprotein E (*apoe*), the key regulator of TG transport, was observed only in L17 group (Fig. 3h). The abundance of glycogenic genes, including in pyruvate carboxylase (*pc*), phosphoenolpyruvate carboxykinase (*pepck*) and glucose-6-phosphatase (*g6pase*), were markedly raised both in L7 and L12 group, but declined in L17 group instead (Fig. 3i, j and k).

## Discussion

Among the evolution of most teleost fishes, an accurate and complicated metabolic system has been developed to respond to different nutritional states [41]. As it shown previously, the increased dietary fat usually could enhance the whole-body lipid content in teleost [42, 43]. Meanwhile, the increase of dietary fat has been demonstrated to exert a protein-sparing effect and improve growth performance in most aquaculture fishes, especially in carnivorous species [7, 8, 44]. Chinese perch, as a typical carnivorous fish, exhibited the slightly elevated growth rate and protein retention from L2 to L12 group, which suggested that dietary fat within certain limit was acted as energy source for better growth performance [6, 10, 11]. Nevertheless, the acute decline in growth rate, food intake and protein retention were observed only in L17 group. Similar results were also found in European sea bass (*Dicentrarchus labrax*) [6], turbot (*Psetta maximus*) [45]. It was indicated that excess fat-intake could result in the poor growth performance, anorexia symptoms and even lipid metabolic disorders [46, 47]. Moreover, it also induced fish stored the extra energy in the form of neutral lipid (mainly TG) in liver, skeletal muscle and viscera cavity [6, 16, 18–21, 48]. Since mass TG has deposited in adipose tissue, it would lose the function of providing energy before the consumption of protein [10, 11]. Regarding the priority of protein utilization in fish, a series of energy dissipation using protein would impair the protein-sparing effect.

**Fig. 4:**
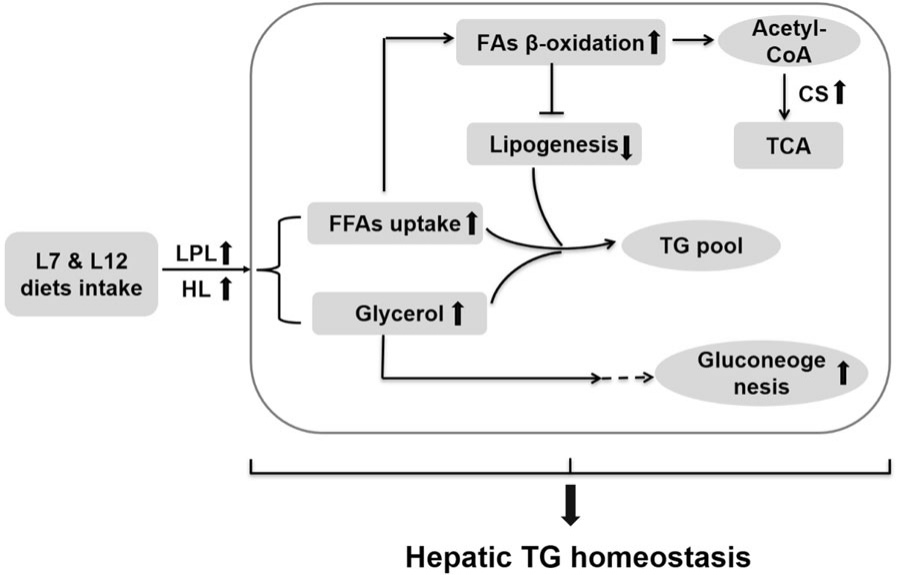
Proposed model of lipid metabolic strategy in Chinese perch responding to appropriate fat-intake. L7 and L12 represent diets containing of 7 and 12% lipid content, which was the optimal range of lipid level for fish growth and utilization in the present study. In the liver, the elevated FFAs-uptake could trigger FAs β-oxidation and then inhibit the lipogenesis to maintain stabilization of TG pool. The Acetyl-CoA derived from FAs β-oxidation could participate in TCA cycle for providing energy. Indeed, the substrate-glycerol derived from dietary TG hydrolysis would participate in gluconeogenesis and then prevent TG from accumulating in the liver. In this model, the homeostasis of hepatic TG pool and serum glucose was through the cooperation of FAs β-oxidation and gluconeogenesis

Within certain limit, the variation of dietary fat level from 2 to 12%, could not influence the homeostasis of hepatic lipid according to the TG content in liver and serum. Similar results had been reported in other fishes, like grass carp [10, 14, 17], Nile tilapia [12], Atlantic halibut (*Hippoglossus hippolossus*) [16], sea bream (*Diplodus sargus*) [49] and meagre (*Argyrosomus regius*) [4]. Meanwhile, the histomorphology of hepatocyte showed that small vacuoles and lipid droplets were emerged progressively in L7 and L12 group, which demonstrated that plasma lipid could pass in liver frequently through esterification of FFAs [50, 51]. But the amount of cell nuclei and relative area of lipid droplet exhibited no statistical difference among the L2, L7 and L12 group. It meant that the extra uptake of FFAs cannot be esterified into TG form and accumulated in hepatocyte. In order to expound the mechanism of lipid metabolism in liver, the expression of several key genes which participated in lipid (*lpl*, *hl*, *cpt1*, *cs*, *srebp1*, *accα*, *fas* and *apoe*) and glucose metabolism (*pc*, *pepck* and *g6pase*) were measured. The adaptive strategy responding to an appropriate fat-intake in the liver was briefly illustrated (Fig. 4). Compared to L2 group, higher fat-intake in L7 and L12 group would accelerate dietary triglyceride hydrolysis and then produce more FFAs- and glycerol-substrate in liver. Higher FFAs-uptake could trigger the FAs β-oxidation via *cpt1*, which cooperated with an overexpression of *cs*, suggesting that the process of TCA cycle was expedited with more acetyl-CoA. The oxidation of acetyl-CoA in TCA cycle could produce carbon dioxide and chemical energy in the form of ATP [52]. Indeed, the increase of fat-intake could also promote the conversion from triglyceride hydrolysis to glycogen synthesis de novo. It was mainly due to that more substrate-glycerol could participate in gluconeogenesis once again via the TCA cycle. These major steps had been taken not only to maintain the concentration of serum glucose but also to prevent mass FFAs from being esterified into TG in liver [22]. The decrease of lipogenesis in liver was mainly due to that it existed a dynamic balance between FAs β-oxidation and lipogenesis [12, 53, 54]. This model would be better for the homeostasis of TG pool and the stabilization of physiological status of Chinese perch.

In addition, it was noticed that the whole-body lipid content of Chinese perch was gradually raised from L2 to L12 group, but the TG content in liver and muscle was stable, on the other hand, both the TG content in visceral adipose tissue and the VSI were increased markedly. Dietary fat (primarily TG) would be digested and absorbed in intestine, and then hydrolyzed in the lumen of the gut by pancreatic lipases, finally formed into FFAs. The FFAs were contiguously transported by lipoproteins lipase in blood and absorbed into liver or adipose tissue for esterification [50, 51]. In this study, it was suggested that visceral adipose tissue could be a priority site for the whole-body lipid deposition in L7 and L12 group. Similar results have been reported in previous studies [4, 10, 12, 14, 16, 17]. It was further suggested that the homeostasis of hepatic lipid was partly due to the visceral adipose tissue participated in storing TG as well.

**Fig. 5:**
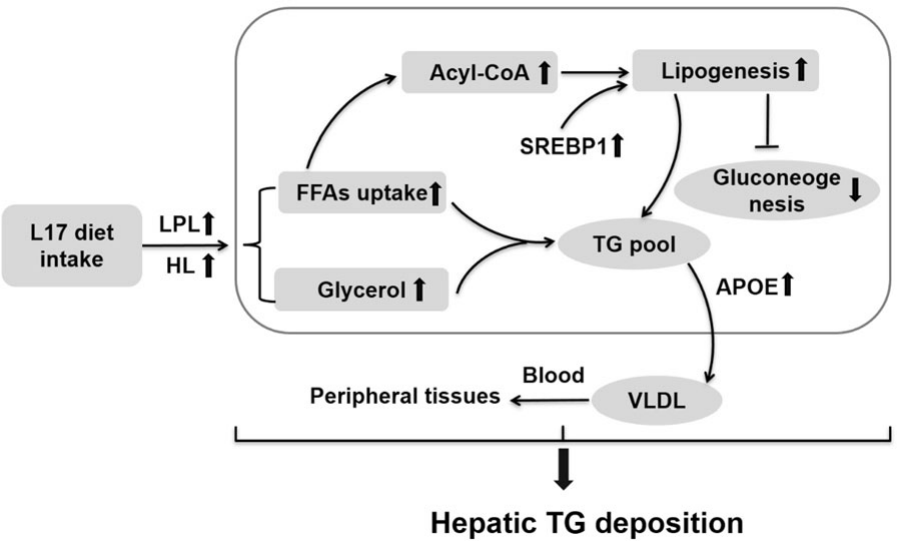
Proposed model of hepatic TG deposition in Chinese perch responding to high-fat intake. L17 represents diets containing of 17% lipid content, which was not benefit for hepatic lipid utilization and metabolism in the present study. In the liver, excess dietary fat-intake could accelerate the esterification of FFAs with the absence of FAs β-oxidation. Indeed, the extra uptake of FFAs could provide the mass Acyl-CoA substrate for lipogenesis via the up-regulation of *srebp1*. The sharply increased lipogenesis could effectively inhibit gluconeogenesis and aggravate TG deposition in liver although a part of newly-synthesized TG had been delivered to peripheral tissues via VLDL in blood

It is authenticated that high-fat diets could contribute to lipid accumulation in the whole body of teleost fishes, but the sites of fat deposition are highly species-specific. These previous studies have been approved that some fishes could store lipid mainly in liver, like cod [9], but salmon could store high amount of lipid mainly in muscle fibers [55]. Preliminary studies indicated that the whole-body lipid content of Chinese perch was elevated with the increased dietary fat from 12 to 17%, but both the VSI and the visceral TG content could not raise contrarily. Surprisingly, both the HSI and the hepatic TG content were significant higher in L17 group. The relative area of lipid droplets and the size of vacuoles in hepatocyte were increased and enlarged separately in L17 group. That means ectopic fat deposition and hepatic steatosis in hepatocyte [56]. Moreover, the higher concentrations of TG, TC, LDL and HDL in blood suggested that the TG-rich metabolites were fast transported by the blood circulation between liver and peripheral tissue. The higher enzymic activity of AST indicated that hepatic injury was caused by TG infiltration in hepatocyte [57]. Similar reports have confirmed that excess fat-intake would induce TG deposition and cause hepatic impairment in some aquaculture fish species [26, 55, 58, 59]. The related mechanism of lipid deposition in liver with high-fat diet is illustrated (Fig. 5). In short, compared to L12 group, higher FFAs-uptake in L17 group would speed up the esterification of acyl-CoA with the absence of FAs β-oxidation. It means that mass FFAs could not participate in mitochondrial oxidation for providing energy via TCA cycle. In contrast, it supplied more substrate of acyl-CoA to trigger lipogenesis via the up-regulation of *srebp1* [53]. Meanwhile, the elevated lipogenesis could also inhibit the progress of gluconeogenesis, and then shut down the conversion from lipid to glycogen (seen in lower serum glucose). Although the newly-synthesized TG could be transported into peripheral tissues via VLDL (*apoe*) in bloodstream (seen in higher serum TC, TG and LDL) [14], it still could not alleviate the accumulation of TG in liver and the hepatic injury. It was confirmed that high-fat diet could induce the lipid metabolic disorders through the shutdown of FAs-oxidation and the unusual aggravation of lipogenesis in liver [29, 57]. In addition, the sharply decreased gluconeogenesis might be the additional factor of hepatic TG deposition [60]. In summary, the hepatic steatosis and injury were mainly due to the mass deposition of newly-synthesized TG, which was caused by the absence of lipolysis and the aggravation of lipogenesis.

## Conclusion

In conclusion, with the increase of dietary fat-intake in L7 and L12 group, the extra uptake of FFAs and glycerol derived from triglyceride hydrolysis would accelerate the FAs β-oxidation and the gluconeogenesis respectively. The negative regulation of FAs β-oxidation could effectively depress lipogenesis, and then shut down the esterification of FFAs. These procedures were critical to maintain the homeostasis of hepatic TG pool and serum glucose, indeed, improve growth performance and reduce the consumption of dietary protein. However, high-fat diet in L17 group could easily impair hepatic lipid homeostasis and induce lipid metabolic disorder, which was caused by the absence of lipolysis and the aggravation of lipogenesis. In addition, the inhibition of gluconeogenesis could also aggravate the TG deposition in liver and then induce the hepatic steatosis. Both the hepatic injury and the decrease of serum glucose could impair the normal physiological status and slow the growth rate in Chinese perch. Overall, our study unraveled the main inducement between the impaired lipid homeostasis and the aggravated lipid deposition in liver, and it might provide implications for the investigation of fatty liver syndrome in teleost fishes.

## Abbreviations

ACCα: Acetyl-CoA carboxylase alpha
APOE: Apo-lipoprotein E
AST: Aspartate aminotransferase
CPT1: Carnitine palmitoyltransferase I
CS: Citrate synthase
EFA: Essential fatty acid
FAS: Fatty acid synthase
FAs: Fatty acids
FFAs: Free fatty acids
FI: Food intake
FW: Final body weight
G6pase: Glucose-6-phosphatase
GLU: Glucose
HDL: High-density lipoprotein
HL: Hepatic lipase
HSI: Hepatic somatic index
IW: Initial body weight
LDL: Low-density lipoprotein
LPL: Lipoprotein lipase
MFI: Mesentery fat index
PC: Pyruvate carboxylase
PEPCK: Phosphoenolpyruvate carboxykinase
PR: Protein retention
SGR: Specific growth ratio
SREBP1: Sterol regulatory element binding protein 1
TC: cholesterol
TCA: Tricarboxylic acid cycle
TG: Triglyceride
VLDL: Very-low density lipoprotein
VSI: Visceral somatic index
WAT: White adipose tissue
WG: Weight gain

## References

[1] Alves Martins D, Rocha F, Martinez-Rodriguez G, Bell G, Morais S, Castanheira F, Bandarra N, Coutinho J, Yufera M, Conceicao LE (2012). Teleost fish larvae adapt to dietary arachidonic acid supply through modulation of the expression of lipid metabolism and stress response genes. Br J Nutr.

[2] Arzel J, Lopez FXM, Metailler R, Stephan G, Viau M, Gandemer G, Guillaume J (1994). Effect of dietary-lipid on growth-performance and body-composition of Brown trout (*Salmo Trutta*) reared in seawater. Aquaculture.

[3] Boujard T, Gelineau A, Coves D, Corraze G, Dutto G, Gasset E, Kaushik S (2004). Regulation of feed intake, growth, nutrient and energy utilisation in European sea bass (*Dicentrarchus labrax*) fed high fat diets. Aquaculture.

[4] Chatzifotis S, Panagiotidou M, Papaioannou N, Pavlidis M, Nengas I, Mylonas CC (2010). Effect of dietary lipid levels on growth, feed utilization, body composition and serum metabolites of meagre (*Argyrosomus regius*) juveniles. Aquaculture.

[5] Chou B-S, Shiau S-Y (1996). Optimal dietary lipid level for growth of juvenile hybrid tilapia, *Oreochromis niloticus* X *Oreochromis aureus*. Aquaculture.

[6] Peres H, Oliva-Teles A (1999). Effect of dietary lipid level on growth performance and feed utilization by European sea bass juveniles (*Dicentrarchus labrax*). Aquaculture.

[7] Beamish FWH, Medland TE (1986). Protein sparing effects in large rainbow-trout, *Salmo Gairdneri*. Aquaculture.

[8] Watanabe T (1982). Lipid nutrition in fish. Comp Biochem Physiol B: Comp Biochem.

[9] Dos Santos J, Burkow IC, Jobling M (1993). Patterns of growth and lipid deposition in cod (*Gadus morhua* L.) fed natural prey and fish-based feeds. Aquaculture.

[10] Du ZY, Clouet P, Zheng WH, Degrace P, Tian LX, Liu YJ (2006). Biochemical hepatic alterations and body lipid composition in the herbivorous grass carp (*Ctenopharyngodon idella*) fed high-fat diets. Br J Nutr.

[11] Du ZY, Liu YJ, Tian LX, Wang JT, Wang Y, Liang GY (2005). Effect of dietary lipid level on growth, feed utilization and body composition by juvenile grass carp (*Ctenopharyngodon idella*). Aquac Nutr.

[12] He An-Yuan, Ning Li-Jun, Chen Li-Qiao, Chen Ya-Li, Xing Qi, Li Jia-Min, Qiao Fang, Li Dong-Liang, Zhang Mei-Ling, Du Zhen-Yu (2015). Systemic adaptation of lipid metabolism in response to low- and high-fat diet in Nile tilapia (Oreochromis niloticus ). Physiological Reports.

[13] HEMRE, SANDNES (1999). Effect of dietary lipid level on muscle composition in Atlantic salmon Salmo salar. Aquaculture Nutrition.

[14] Li AX, Yuan XC, Liang XF, Liu LW, Li J, Li B, Fang JG, Li J, He S, Xue M (2016). Adaptations of lipid metabolism and food intake in response to low and high fat diets in juvenile grass carp (*Ctenopharyngodon idellus*). Aquaculture.

[15] Lopez LM, Durazo E, Viana MT, Drawbridge M, Bureau DP (2009). Effect of dietary lipid levels on performance, body composition and fatty acid profile of juvenile white seabass, *Atractoscion nobilis*. Aquaculture.

[16] Martins DA, Valente LMP, Lall SP (2007). Effects of dietary lipid level on growth and lipid utilization by juvenile Atlantic halibut (*Hippoglossus hippoglossus*, L.). Aquaculture.

[17] Yuan XC, Liang XF, Liu LW, Fang JG, Li J, Li AX, Cai WJ, Xue M, Wang J, Wang QC (2016). Fat deposition pattern and mechanism in response to dietary lipid levels in grass carp, *Ctenopharyngodon idellus*. Fish Physiol Biochem.

[18] Pei Z, Xie S, Lei W, Zhu X, Yang Y (2004). Comparative study on the effect of dietary lipid level on growth and feed utilization for gibel carp (*Carassius auratus gibelio*) and Chinese longsnout catfish (*Leiocassis longirostris Günther*). Aquac Nutr.

[19] Regost C, Arzel J, Cardinal M, Robin J, Laroche M, Kaushik SJ (2001). Dietary lipid level, hepatic lipogenesis and flesh quality in turbot (*Psetta maxima*). Aquaculture.

[20] Song LP, An LG, Zhu YA, Li X, Wang AY (2009). Effects of dietary lipids on growth and feed utilization of jade perch, *Scortum barcoo*. J World Aquacult Soc.

[21] Wang JT, Liu YJ, Tian LX, Mai KS, Du ZY, Wang Y, Yang HJ (2005). Effect of dietary lipid level on growth performance, lipid deposition, hepatic lipogenesis in juvenile cobia (*Rachycentron canadum*). Aquaculture.

[22] Morash AJ, Bureau DP, McClelland GB (2009). Effects of dietary fatty acid composition on the regulation of carnitine palmitoyltransferase (CPT) I in rainbow trout (*Oncorhynchus mykiss*). Comp Biochem Physiol B Biochem Mol Biol.

[23] Du Z-Y, Ma T, Liaset B, Keenan AH, Araujo P, Lock E-J, Demizieux L, Degrace P, Frøyland L, Kristiansen K, Madsen L (2013). Dietary eicosapentaenoic acid supplementation accentuates hepatic triglyceride accumulation in mice with impaired fatty acid oxidation capacity. Biochim Biophys Acta.

[24] Unger RH (2003). The physiology of cellular liporegulation. Annu Rev Physiol.

[25] Xu JH, Qin J, Yan BL, Zhu M, Luo G (2011). Effects of dietary lipid levels on growth performance, feed utilization and fatty acid composition of juvenile Japanese seabass (*Lateolabrax japonicus*) reared in seawater. Aquac Int.

[26] Nanton DA, Lall SP, McNiven MA (2001). Effects of dietary lipid level on liver and muscle lipid deposition in juvenile haddock, *Melanogrammus aeglefinus* L. Aquac Res.

[27] Savage DB, Petersen KF, Shulman GI (2007). Disordered lipid metabolism and the pathogenesis of insulin resistance. Physiol Rev.

[28] Jocken JWE, Langin D, Smit E, Saris WHM, Valle C, Hul GB, Holm C, Arner P, Blaak EE (2007). Adipose triglyceride lipase and hormone-sensitive lipase protein expression is decreased in the obese insulin-resistant state. J Clin Endocrinol Metab.

[29] Khasawneh J, Schulz MD, Walch A, Rozman J, Hrabe de Angelis M, Klingenspor M, Buck A, Schwaiger M, Saur D, Schmid RM (2009). Inflammation and mitochondrial fatty acid beta-oxidation link obesity to early tumor promotion. Proc Natl Acad Sci U S A.

[30] Chen Y, Varghese Z, Ruan XZ (2014). The molecular pathogenic role of inflammatory stress in dysregulation of lipid homeostasis and hepatic steatosis. Genes Dis.

[31] Liang XF, Oku H, Ogata HY, Liu J, He X (2001). Weaning Chinese perch *Siniperca chuatsi* (Basilewsky) onto artificial diets based upon its specific sensory modality in feeding. Aquac Res.

[32] Strebkova TP, Shabalina VN. Farming of Chinese perch[J]. Marine Fish Culture. 1984:41–52.

[33] Liang XF (2002). Study on Chinese perch (*Siniperca chuatsi*) artificial diets. Fish Sci Technol Inform.

[34] Liang XF. Study on Manderin fish and its culture home and abroad. Fish Sci Technol Inform. 1996.

[35] Horwitz W (1995). Official methods of analysis of the Association of Official Analytical Chemists. J Pharm Sci.

[36] Chen HC, Farese RV (2002). Determination of adipocyte size by computer image analysis. J Lipid Res.

[37] Hopwood D (1977). Histopathologic technic and practical Histochemistry (4th edition). Biochem Soc Trans.

[38] He S, Liang XF, Sun J, Li L, Yu Y, Huang W, Qu CM, Cao L, Bai XL, Tao YX (2013). Insights into food preference in hybrid F1 of *Siniperca chuatsi* (female symbol) x *Siniperca scherzeri* (male symbol) mandarin fish through transcriptome analysis. BMC Genomics.

[39] Vandesompele Jo, De Preter Katleen, Pattyn Filip, Poppe Bruce, Van Roy Nadine, De Paepe Anne, Speleman Frank (2002). Genome Biology.

[40] Livak KJ, Schmittgen TD (2001). Analysis of relative gene expression data using real-time quantitative PCR and the 2(−Delta Delta C(T)) method. Methods.

[41] Soengas JL (2014). Contribution of glucose- and fatty acid sensing systems to the regulation of food intake in fish. A review. Gen Comp Endocrinol.

[42] Achman RG (1995). Composition and nutritive value of fish and shellfish lipids. Fish & Fishery Products.

[43] Robb DHF, Kestin SC, Warriss PD, Nute GR (2002). Muscle lipid content determines the eating quality of smoked and cooked Atlantic salmon (*Salmo salar*). Aquaculture.

[44] Dias J, Alvarez MJ, Diez A, Arzel J, Corraze G, Bautista JM, Kaushik SJ (1998). Regulation of hepatic lipogenesis by dietary protein/energy in juvenile European seabass (*Dicentrarchus labrax*). Aquaculture.

[45] Saether BS, Jobling M (2001). Fat content in turbot feed: influence on feed intake, growth and body composition. Aquac Res.

[46] Takeuchi T, Toyota M, Satoh S, Watanabe T (1990). Requirement of juvenile red seabream *Pagrus major* for eicosapentaenoic and docosahexaenoic acids. Nippon Suisan Gakkaishi.

[47] Watanabe T (1993). Importance of docosahexaenoic acid in marine larval fish. J World Aquacult Soc.

[48] Ghanawi J, Roy L, Davis DA, Saoud IP (2011). Effects of dietary lipid levels on growth performance of marbled spinefoot rabbitfish *Siganus rivulatus*. Aquaculture.

[49] Sa R, Pousao-Ferreira P, Oliva-Teles A (2006). Effect of dietary protein and lipid levels on growth and feed utilization of white sea bream (*Diplodus sargus*) juveniles. Aquac Nutr.

[50] Sheridan MA (1988). Lipid dynamics in fish: aspects of absorption, transportation, deposition and mobilization. Comp Biochem Physiol B.

[51] Tocher DR (2003). Metabolism and functions of lipids and fatty acids in teleost fish. Rev Fish Sci.

[52] Wiegand G, Remington SJ (1986). Citrate synthase: structure, control, and mechanism. Annu Rev Biophys Biophys Chem.

[53] Leng XJ, Wu XF, Tian J, Li XQ, Guan L, Weng DC (2012). Molecular cloning of fatty acid synthase from grass carp (*Ctenopharyngodon idella*) and the regulation of its expression by dietary fat level. Aquac Nutr.

[54] Rollin X, Medale F, Gutieres S, Blanc D, Kaushik SJ (2003). Short- and long-term nutritional modulation of acetyl-CoA carboxylase activity in selected tissues of rainbow trout (*Oncorhynchus mykiss*). Br J Nutr.

[55] Torstensen BE, Lie O, Hamre K (2001). A factorial experimental design for investigation of effects of dietary lipid content and pro- and antioxidants on lipid composition in Atlantic salmon (*Salmo salar*) tissues and lipoproteins. Aquac Nutr.

[56] Wei CC, Wu K, Gao Y, Zhang LH, Li DD, Luo Z (2017). Magnesium reduces hepatic lipid accumulation in yellow catfish (*Pelteobagrus fulvidraco*) and modulates lipogenesis and lipolysis via PPARA, JAK-STAT, and AMPK Pathways in Hepatocytes. J Nutr.

[57] Meli R, Mattace Raso G, Irace C, Simeoli R, Di Pascale A, Paciello O, Pagano TB, Calignano A, Colonna A, Santamaria R (2013). High fat diet induces liver steatosis and early dysregulation of Iron metabolism in rats. PLoS One.

[58] Gaylord TG, Gatlin DM (2000). Dietary lipid level but not l-carnitine affects growth performance of hybrid striped bass (Morone chrysops ♀×M. saxatilis ♂). Aquaculture.

[59] Lu KL, Xu WN, Li XF, Liu WB, Wang LN, Zhang CN (2013). Hepatic triacylglycerol secretion, lipid transport and tissue lipid uptake in blunt snout bream (*Megalobrama amblycephala*) fed high-fat diet. Aquaculture.

[60] Panserat S, Plagnes-Juan E, Kaushik S (2002). Gluconeogenic enzyme gene expression is decreased by dietary carbohydrates in common carp (*Cyprinus carpio*) and gilthead seabream (*Sparus aurata*). Biochim Biophys Acta.

